# Supplementary material for: Characterizing a thermostable Cas9 for bacterial genome editing and silencing
Source: Nat Commun. 2017 Nov 21;8:1647. doi: 10.1038/s41467-017-01591-4 (PMC5698299; doi:10.1038/s41467-017-01591-4)
Supplement: Supplementary file 1 — Supplementary Information [file 41467_2017_1591_MOESM1_ESM.pdf]

Supplementary Table 1 | pBLAST results of Cas9 protein sequences from Figure 1B compared to ThermoCas9.

**Species**	**% identity[a]**
Geobacillus 47C-IIb	99
Geobacillus 46C-IIa	89
Geobacillus LC300	89
Geobacillus jurassicus	89
Geobacillus MAS1	88
Geobacillus stearothermophilus	88
Geobacillus stearothermophilus ATCC 12980	88
Geobacillus Sah69	88
Geobacillus stearothermophilus	88
Geobacillus kaustophilus	88
Geobacillus stearothermophilus	88
Geobacillus genomosp. 3	87
Geobacillus genomosp. 3	87
Geobacillus subterraneus	87
Effusibacillus pohliae	86

[a] Query coverage was 100% in all cases.

Supplementary Table 2 | Oligonucleotides used in this study, related to Figures 1 to 4.

	Oligo	Sequence	Description
**PAM Library construction**	BG6494	TATGCC***TCATGA*GATTATCAAAAAGGATCTTCAC**NNNNNNNCTAGATCCTTTTAAATTAAAAATGAAGTTTTAAATCAATC	FW for construction of *in vitro* target DNA with 7-nt long random PAM sequence
	BG6495	TATGCC***GGATCC***TCAGACCAAGTTTACTCATATATACTTTAGATTGATTTAAAACTTCATTTTTAATTTAAAAGGATCTAG	RV for construction of *in vitro* target DNA sequences
	BG7356	TCGTCGGCAGCGTCAGATGTGTATAAGAGACAG-T-	Adaptor when annealed with BG7357, ligates to A-tailed ThermoCas9 cleaved fragments
	BG7357	CTGTCTCTTATACACATCTGACGCTGCCGACGA	Adaptor when annealed with BG7356, ligates to A-tailed ThermoCas9 cleaved fragments
	BG7358	TCGTCGGCAGCGTCAG	FW sequencing adaptor for PCR amplification of the ThermoCas9 cleaved fragments
	BG7359	GTCTCGTGGGCTCGGAGATGTGTATAAGAGACAGGACCATGATTACGCCAAGC	RV sequencing adapter for PCR amplification of the ThermoCas9 cleaved fragments
	BG7616	TCGTCGGCAGCGTCAGATGTGTATAAGAGACAGGGTCATGAGATTATCAAAAAGGATCTTC	RV sequencing adaptor for PCR amplification of the control fragments
	BG8157	TATGCC***TCATGA*GATTATCAAAAAGGATCTTCACCCCCCCAG**CTAGATCCTTTTAAATTAAAAATGAAGTTTTAAATCAATC	FW for construction of *in vitro* target DNA with PAM "CCCCCCAG"
	BG8158	TATGCC**TCATGAGATTATCAAAAAGGATCTTCACCCCCCCAA**CTAGATCCTTTTAAATTAAAAATGAAGTTTTAAATCAATC	FW for construction of *in vitro* target DNA with PAM "CCCCCCAA"
	BG8159	**TATGCC*TCATGA*GATTATCAAAAAGGATCTTCACCCCCCCAT**CTAGATCCTTTTAAATTAAAAATGAAGTTTTAAATCAATC	FW for construction of *in vitro* target DNA with PAM "CCCCCCAT"
	BG8160	TATGCC***TCATGA*GATTATCAAAAAGGATCTTCACCCCCCCAC**CTAGATCCTTTTAAATTAAAAATGAAGTTTTAAATCAATC	FW for construction of *in vitro* target DNA with PAM "CCCCCCAC"
	BG8161	TATGCC***TCATGA*GATTATCAAAAAGGATCTTCACNNNNTNN**CTAGATCCTTTTAAATTAAAAATGAAGTTTTAAATCAATC	FW for construction of *in vitro* target DNA with PAM "NNNNTNN"
	BG8363	ACGGTTATCCACAGAATCAG	FW for PCR linearization of PAM identification libraries
	BG8364	CGGGATTGACTTTTAAAAAAGG	RV for PCR linearization of PAM identification libraries
	BG8763	TATGCC***TCATGA*GATTATCAAAAAGGATCTTCACCCCCCAAA**CTAGATCCTTTTAAATTAAAAATGAAGTTTTAAATCAATC	FW for construction of *in vitro* target DNA with PAM position 6&7 "AA"
	BG8764	**TATGCC*TCATGA*GATTATCAAAAAGGATCTTCACCCCCCATA**CTAGATCCTTTTAAATTAAAAATGAAGTTTTAAATCAATC	FW for construction of *in vitro* target DNA with PAM position 6&7 "AT"
	BG8765	TATGCC***TCATGA*GATTATCAAAAAGGATCTTCACCCCCCAGA**CTAGATCCTTTTAAATTAAAAATGAAGTTTTAAATCAATC	FW for construction of in *vitro* target DNA with PAM position 6&7 "AG"
	BG8766	TATGCC***TCATGA*GATTATCAAAAAGGATCTTCACCCCCCACA**CTAGATCCTTTTAAATTAAAAATGAAGTTTTAAATCAATC	FW for construction of *in vitro* target DNA with PAM position 6&7 "AC"

	Oligo	Sequence	Description
**PAM Library construction**	BG8767	TATGCC***TCATGA*****GATTATCAAAAAGGATCTTCAC**CCCCCTAACTAG ATCCTTTTAAATTAAAAATGAAGTTTTAAATCAATC	FW for construction of *in vitro* target DNA with PAM position 6&7 "TA"
	BG8768	TATGCC***TCATGA*****GATTATCAAAAAGGATCTTCAC**CCCCCTTACTAG ATCCTTTTAAATTAAAAATGAAGTTTTAAATCAATC	FW for construction of *in vitro* target DNA with PAM position 6&7 "TT"
	BG8769	TATGCC***TCATGA*****GATTATCAAAAAGGATCTTCAC**CCCCCTGACTAG ATCCTTTTAAATTAAAAATGAAGTTTTAAATCAATC	FW for construction of *in vitro* target DNA with PAM position 6&7 "TG"
	BG8770	TATGCC***TCATGA*****GATTATCAAAAAGGATCTTCAC**CCCCCTCACTAG ATCCTTTTAAATTAAAAATGAAGTTTTAAATCAATC	FW for construction of *in vitro* target DNA with PAM position 6&7 "TC"
	BG8771	TATGCC***TCATGA*****GATTATCAAAAAGGATCTTCAC**CCCCCGAACTAG ATCCTTTTAAATTAAAAATGAAGTTTTAAATCAATC	FW for construction of *in vitro* target DNA with PAM position 6&7 "GA"
	BG8772	TATGCC***TCAT*****GAGATTATCAAAAAGGATCTTCAC**CCCCCGTACTAG ATCCTTTTAAATTAAAAATGAAGTTTTAAATCAATC	FW for construction of *in vitro* target DNA with PAM position 6&7 "GT"
	BG8773	TATGCC***TCATGA*****GATTATCAAAAAGGATCTTCAC**CCCCCGGACTAG ATCCTTTTAAATTAAAAATGAAGTTTTAAATCAATC	FW for construction of *in vitro* target DNA with PAM position 6&7 "GG"
	BG8774	TATGCC***TCATGA*****GATTATCAAAAAGGATCTTCAC**CCCCCGCACTAG ATCCTTTTAAATTAAAAATGAAGTTTTAAATCAATC	FW for construction of *in vitro* target DNA with PAM position 6&7 "GC"
	BG8775	TATGCC***TCATGA*****GATTATCAAAAAGGATCTTCAC**CCCCCCAACTAG ATCCTTTTAAATTAAAAATGAAGTTTTAAATCAATC	FW for construction of *in vitro* target DNA with PAM position 6&7 "CA"
	BG8776	TATGCC***TCATGA*****GATTATCAAAAAGGATCTTCAC**CCCCCCTACTAG ATCCTTTTAAATTAAAAATGAAGTTTTAAATCAATC	FW for construction of *in vitro* target DNA with PAM position 6&7 "CT"
	BG8777	TATGCC***TCATGA*****GATTATCAAAAAGGATCTTCAC**CCCCCCGACTAG ATCCTTTTAAATTAAAAATGAAGTTTTAAATCAATC	FW for construction of *in vitro* target DNA with PAM position 6&7 "CG"
	BG8778	TATGCC***TCATGA*****GATTATCAAAAAGGATCTTCAC**CCCCCCCACTAG ATCCTTTTAAATTAAAAATGAAGTTTTAAATCAATC	FW for construction of *in vitro* target DNA with PAM position 6&7 "CC"
**sgRNA module for *in vitro* transcription**	BG6574	AAGCTTGAAATAATACGACTCACTATAGG	FW for PCR amplification of the sgRNA template for the first PAM identification process (30nt long spacer)
	BG6576	AAAAAAGACCTTGACGTTTTCC	FW for PCR amplification of the sgRNA template for the first PAM identification process
	BG9307	AAGCTTGAAATAATACGACTCACTATAGGTGAGATTATCAAAAAGG ATCTTCACGTC	RV for PCR amplification of the sgRNA template for all the PAM identification processes except the first one (25nt long spacer)
	BG9309	AAAACGCCTAAGAGTGGGGAATG	RV for PCR amplification of the 3-hairpins long sgRNA template for all the PAM identification processes except the first one
	BG9310	AAAAGGCGATAGGCGATCC	RV for PCR amplification of the 2-hairpins long sgRNA template for all the PAM identification processes except the first one
	BG9311	AAAACGGGTCAGTCTGCCTATAG	RV for PCR amplification of the 1-hairpin long sgRNA template for all the PAM identification processes except the first one
	BG9308	AAGCTTGAAATAATACGACTCACTATAGGTGAGATTATCAAAAAGG ATCTTCACGTC	pT7 and 25nt spacer sgRNA Fw

	Oligo	Sequence	Description
**sgRNA module for *in vitro* transcription**	BG10118	AAGCTTGAAATAATACGACTCACTATAGGAGATTATCAAAAAGGATCTTCACGTCA	pT7 and 24nt spacer sgRNA Fw
	BG10119	AAGCTTGAAATAATACGACTCACTATAGGAAGATTATCAAAAAGGATCTTCACGTCATAG	pT7 and 23nt spacer sgRNA Fw
	BG10120	AAGCTTGAAATAATACGACTCACTATAGGATTATCAAAAAGGATCTTCACGTCATAGT	pT7 and 22nt spacer sgRNA Fw
	BG10121	AAGCTTGAAATAATACGACTCACTATAGGAATTATCAAAAAGGATCTTCACGTCATAGTT	pT7 and 21nt spacer sgRNA Fw
	BG10122	AAGCTTGAAATAATACGACTCACTATAGGTTATCAAAAAGGATCTTCACGTCATAGTT	pT7 and 20nt spacer sgRNA Fw
	BG10123	AAGCTTGAAATAATACGACTCACTATAGGTATCAAAAAGGATCTTCACGTCATAGTTC	pT7 and 19nt spacer sgRNA Fw
	BG10124	AAGCTTGAAATAATACGACTCACTATAGGATCAAAAAGGATCTTCACGTCATAGTTC	pT7 and 18nt spacer sgRNA Fw
**Editing and silencing constructs**	BG9312	AAAACGCCTAAGAGTGGGGAATGCCCGAAGAAAGCGGGCGATAGGCGATCC	3 loops sgRNA OH Rv
	BG8191	AAGCTTGGCGTAATCATGGTC	For the construction of the pThermoCas9_ctrl plasmid & pThermoCas9_bsΔpyrF1/2
	BG8192	TCATGAGTTCCCATGTTGTG	For the construction of the pThermoCas9_ctrl plasmid & pThermoCas9_bsΔpyrF1/2
	BG8194	tatggcgaatcacaacatgggaactcatgaGAACATCCTCTTTCTTAG	For the construction of the pThermoCas9_ctrl plasmid & pThermoCas9_bsΔpyrF1/2
	BG8195	gccgatatcaagaccgattttatacttcatTTAAGTTACCTCCTCGATTG	For the construction of the pThermoCas9_ctrl plasmid & pThermoCas9_bsΔpyrF1/2
	BG8196	ATGAAGTATAAAATCGGTCTTG	For the construction of the pThermoCas9_ctrl plasmid & pThermoCas9_bsΔpyrF1/2
	BG8197	TAACGGACGGATAGTTTC	For the construction of the pThermoCas9_ctrl plasmid & pThermoCas9_bsΔpyrF1/2
	BG8198	gaaagccggggaaactatccgtccgttataAATCAGACAAAATGGCCTGCTTATG	For the construction of the pThermoCas9_ctrl plasmid & pThermoCas9_bsΔpyrF1/2
	BG8263	gaactatgacactttattttcagaatggacGTATAACGGTATCCATTTTAAGAATAATCC	For the construction of the pThermoCas9_ctrl plasmid
	BG8268	accgttatacgtccattctgaaaataaagtGTCATAGTTCCCCTGAGAT	For the construction of the pThermoCas9_ctrl plasmid
	BG8210	aacagctatgaccatgattacgccaagcttCCCTCCCATGCACAATAG	For the construction of the pThermoCas9_ctrl plasmid & pThermoCas9_bsΔpyrF1/2
	BG8261	gaactatgacatcatggagttttaaatccaGTATAACGGTATCCATTTTAAGAATAATCC	For the construction of the pThermoCas9_bsΔpyrF1
	BG8266	accgttatactggatttaaaactccatgatGTCATAGTTCCCCTGAGAT	For the construction of the pThermoCas9_bsΔpyrF2
	BG8317	gaactatgaccacccagcttacatcaacaaGTATAACGGTATCCATTTTAAGAATAATCC	For the construction of the pThermoCas9_ΔbspyrF2
	BG8320	accgttatacttgttgatgtaagctgggtgGTCATAGTTCCCCTGAGAT	For the construction of the pThermoCas9_bsΔpyrF2

	Oligo	Sequence	Description
**Editing and silencing constructs**	BG9075	CTATCGGCATTACGTCTATC	For the construction of the pThermoCas9i_ctrl
	BG9076	GCGTCGACTTCTGTATAGC	For the construction of the pThermoCas9i_ctrl
	BG9091	TGAAGTATAAAATCGGTCTTGCTATCGGCATTACGTCTATC	For the construction of the pThermoCas9i_ctrl
	BG9092	CAAGCTTCGGCTGTATGGAATCACAGCGTCGACTTCTGTATAGC	For the construction of the pThermoCas9i_ctrl
	BG9077	GCTGTGATTCCATACAG	For the construction of the pThermoCas9i_ctrl
	BG9267	GGTGCAGTAGGTTGCAGCTATGCTTGTATAACGGTATCCAT	For the construction of the pThermoCas9i_ctrl
	BG9263	AAGCATAGCTGCAACCTACTGCACCGTCATAGTTCCCCTGAGATTATCG	For the construction of the pThermoCas9i_ctrl
	BG9088	TCATGACCAAAATCCCTTAACG	For the construction of the pThermoCas9i_ctrl
	BG9089	TTAAGGGATTTTGGTCATGAGAACATCCTCTTTCTTAG	For the construction of the pThermoCas9i_ctrl
	BG9090	GCAAGACCGATTTTATACTTCATTTAAG	For the construction of the pThermoCas9i_ctrl
	BG9548	GGATCCCATGACGCTAGTATCCAGCTGGGTCATAGTTCCCCTGAGATTATCG	For the construction of the pThermoCas9i_ldhL
	BG9601	TTCAATATTTTTTTTGAATAAAAAAATACGATACAATAAAAATGTCTAGAAAAAGATAAAAATG	For the construction of the pThermoCas9i_ldhL
	BG9600	TTTTTTATTCAAAAAAAATATTGAATTTTAAAAATGATGGTGCTAGTATGAAG	For the construction of the pThermoCas9i_ldhL
	BG9549	CCAGCTGGATACTAGCGTCATGGGATCCGTATAACGGTATCCATTTTAAGAATAATCC	For the construction of the pThermoCas9i_ldhL
	BG8552	TCGGGGGTTCGTTTCCCTTG	FW to check genomic *pyrF* deletion KO check
	BG8553	CTTACACAGCCAGTGACGGAAC	RV to check genomic *pyrF* deletion KO check
	BG2365	GCCGGCGTCCCGGAAAACGA	For the construction of the pThermoCas9_ppΔpyrF
	BG2366	GCAGGTCGGGTTCCTCGCATCCATGCCCCCGAACT	For the construction of the pThermoCas9_ppΔpyrF
	BG2367	ggcttcggaatcgttttccgggacgccggcACGGCATTGGCAAGGCCAAG	For the construction of the pThermoCas9_ppΔpyrF
	BG2368	gacacaggcatcggtGCAGGGTCTCTTGGCAAGTC	For the construction of the pThermoCas9_ppΔpyrF
	BG2369	gccaagagaccctgCACCGATGCCTGTGTCGAACC	For the construction of the pThermoCas9_ppΔpyrF
	BG2370	cttggcggaaaacgtcaaggtcttttttacACGCGCATCAACTTCAAGGC	For the construction of the pThermoCas9_ppΔpyrF
	BG2371	atgac**gagctgttcaccagcagcgc**TATTATTGAAGCATTTATCAGGG	For the construction of the pThermoCas9_ppΔpyrF
	BG2372	GTAAAAAAGACCTTGACGTTTTC	For the construction of the pThermoCas9_ppΔpyrF

	Oligo	Sequence	Description
**Editing and silencing constructs**	BG2373	tatgaagcgggccatTTGAAGACGAAAGGGCCTC	For the construction of the pThermoCas9_ppΔpyrF
	BG2374	taata**gcgctgctggtgaacagctc**GTCATAGTTCCCCTGAGATTATCG	For the construction of the pThermoCas9_ppΔpyrF
	BG2375	tggagtcatgaacatATGAAGTATAAAATCGGTCTTG	For the construction of the pThermoCas9_ppΔpyrF
	BG2376	ccctttcgtcttcAAATGGCCCGCTTCATAAGCAG	For the construction of the pThermoCas9_ppΔpyrF
	BG2377	gattttatacTTCATATGTTCATGACTCCATTATTATTG	For the construction of the pThermoCas9_ppΔpyrF
	BG2378	gggggcatggatgCGAGGAACCCGACCTGCATTGG	For the construction of the pThermoCas9_ppΔpyrF
	BG2381	ACACGGCGGATGCACTTACC	FW for confirmation of plasmid integration and *pyrF* deletion in *P. putida*
	BG2382	TGGACGTGTACTTCGACAAC	RV for confirmation of *pyrF* deletion in *P. putida*
	BG2135	ACACGGCGGATGCACTTACC	RV for confirmation of plasmid integration in *P. putida*
**Sequencing primers**	BG8196	TGGACGTGTACTTCGACAAC	*thermocas9* seq. 1
	BG8197	TAACGGACGGATAGTTTC	*thermocas9* seq. 2
	BG6850	GCCTCATGAATGCAGCGATGGTCCGGTGTTC	*pyr*F US
	BG6849	GCCTCATGAGTTCCCATGTTGTGATTC	*pyrF* DS
	BG6769	CAATCCAACTGGGCTTGAC	*thermocas9* seq. 3
	BG6841	CAAGAACTTTATTGGTATAG	*thermocas9* seq. 4
	BG6840	TTGCAGAAATGGTTGTCAAG	*thermocas9* seq. 5
	BG9215	GAGATAATGCCGACTGTAC	pNW33n backbone seq. 1
	BG9216	AGGGCTCGCCTTTGGGAAG	pNW33n backbone seq. 2
	BG9505	GTTGCCAACGTTCTGAG	*thermocas9* seq. 6
	BG9506	AATCCACGCCGTTTAG	*thermocas9* seq. 7
**Cleavage assays**	BG8363	ACGGTTATCCACAGAATCAG	FW for PCR linearization of DNA target
	BG8364	CGGGATTGACTTTTAAAAAAGG	RV for PCR linearization of DNA target
	BG9302	AAACTTCATTTTTAATTTAAAAGGATCTAGAACCCCCCGTGAAGATC CTTTTTGATAATCTCATGACCAAAATCCCTTAACGTGAGTTTTCGTTC CACTGAGCGTCAGACCCCGTAGAAA	Non-template strand oligonucleotide for ssDNA cleavage assays
	BG9303	TTTCTACGGGGTCTGACGCTCAGTGGAACGAAAACTCACGTTAAGGG ATTTTGGTCATGAGATTATCAAAAAGGATCTTCACCCCCCCAACTAG ATCCTTTTAAATTAAAAATGAAGTTT	Template strand oligonucleotide for ssDNA cleavage assays

	Oligo	Sequence	Description
	BG9304	TTTCTACGGGGTCTGACGCTCAGTGGAACGAAAACTCACGTTAAGGG ATTTTGGTCATGAGATTATCAAAAAGGATCTTCACGGGGGGTTCTAG ATCCTTTTAAATTAAAAATGAAGTTT	Template strand oligonucleotide for ssDNA cleavage assays
**ThermoCas9 expression**	BG7886	TACTTCCAATCCAATGCAAAGTATAAAATCGGTCTTGATATCG	FW LIC_*thermocas9*
	BG7887	TTATCCACTTCCAATGTTATTATAACGGACGGATAGTTTCCCCGGCTT TC	RV LIC_*thermocas9*
**RT-qPCR**	BG9665	ATGACGAAAGGAGTTTCTTATTATG	RV qPCR check *ldhl*
	BG9666	AACGGTATTCCGTGATTAAG	FW qPCR check *ldhl*
***In vitro* ThermoCas9 Mismatch assay**	BG10561	CATGAGATTATCAAAAAGGATCTTCAACCCCCCAACTAGG	FW for construction of *in vitro* target DNA with mismatch at PAM proximal position 1
	BG10562	GATCCCTAGTTGGGGGGTTGAAGATCCTTTTTGATAATCT	RV for construction of *in vitro* target DNA with mismatch at PAM proximal position 1
	BG10563	CATGAGATTATCAAAAAGGATCTTCCCCCCCCAACTAGG	FW for construction of *in vitro* target DNA with mismatch at PAM proximal position 2
	BG10564	GATCCCTAGTTGGGGGGGGGAAGATCCTTTTTGATAATCT	RV for construction of *in vitro* target DNA with mismatch at PAM proximal position 2
	BG10565	CATGAGATTATCAAAAAGGATCTTAACCCCCCCAACTAGG	FW for construction of *in vitro* target DNA with mismatch at PAM proximal position 3
	BG10566	GATCCCTAGTTGGGGGGGTTAAGATCCTTTTTGATAATCT	RV for construction of *in vitro* target DNA with mismatch at PAM proximal position 3
	BG10567	CATGAGATTATCAAAAAGGATCTGCACCCCCCCAACTAGG	FW for construction of *in vitro* target DNA with mismatch at PAM proximal position 4
	BG10568	GATCCCTAGTTGGGGGGGTGCAGATCCTTTTTGATAATCT	RV for construction of *in vitro* target DNA with mismatch at PAM proximal position 4
	BG10569	CATGAGATTATCAAAAAGGATCGTCACCCCCCCAACTAGG	FW for construction of *in vitro* target DNA with mismatch at PAM proximal position 5
	BG10570	GATCCCTAGTTGGGGGGGTGACGATCCTTTTTGATAATCT	RV for construction of *in vitro* target DNA with mismatch at PAM proximal position 5
	BG10571	CATGAGATTATCAAAAAGGATATTCACCCCCCCAACTAGG	FW for construction of *in vitro* target DNA with mismatch at PAM proximal position 6

	Oligo	Sequence	Description
In vitro **ThermoCas9 Mismatch assay**	BG10572	GATCCCTAGTTGGGGGGGTGAATATCCTTTTTGATAATCT	RV for construction of *in vitro* target DNA with mismatch at PAM proximal position 6
	BG10573	CATGAGATTATCAAAAAGGAGCTTCACCCCCCCAACTAGG	FW for construction of *in vitro* target DNA with mismatch at PAM proximal position 7
	BG10574	GATCCCTAGTTGGGGGGGTGAAGCTCCTTTTTGATAATCT	RV for construction of *in vitro* target DNA with mismatch at PAM proximal position 7
	BG10575	CATGAGATTATCAAAAAGGCTCTTCACCCCCCCAACTAGG	FW for construction of *in vitro* target DNA with mismatch at PAM proximal position 8
	BG10576	GATCCCTAGTTGGGGGGGTGAAGAGCCTTTTTGATAATCT	RV for construction of *in vitro* target DNA with mismatch at PAM proximal position 8
	BG10577	CATGAGATTATCAAAAAGTATCTTCACCCCCCCAACTAGG	FW for construction of *in vitro* target DNA with mismatch at PAM proximal position 9
	BG10578	GATCCCTAGTTGGGGGGGTGAAGATACTTTTTGATAATCT	RV for construction of *in vitro* target DNA with mismatch at PAM proximal position 9
	BG10579	CATGAGATTATCAAAAATGATCTTCACCCCCCCAACTAGG	FW for construction of *in vitro* target DNA with mismatch at PAM proximal position 10
	BG10580	GATCCCTAGTTGGGGGGGTGAAGATCATTTTTGATAATCT	RV for construction of *in vitro* target DNA with mismatch at PAM proximal position10
	BG10581	CATGAGATTATCCAAAAGGATCTTCACCCCCCCAACTAGG	FW for construction of *in vitro* target DNA with mismatch at PAM proximal position 15
	BG10582	GATCCCTAGTTGGGGGGGTGAAGATCCTTTTGGATAATCT	RV for construction of *in vitro* target DNA with mismatch at PAM proximal position 15
	BG10583	CATGAGAGTATCAAAAAGGATCTTCACCCCCCCAACTAGG	FW for construction of *in vitro* target DNA with mismatch at PAM proximal position 20
	BG10584	GATCCCTAGTTGGGGGGGTGAAGATCCTTTTTGATACTCT	RV for construction of *in vitro* target DNA with mismatch at PAM proximal position 20
	BG10585	CATGAGAGGATCAAAAAGGATCTTCACCCCCCCAACTAGG	FW for construction of *in vitro* target DNA with mismatch at PAM distal positions 19-20
	BG10586	GATCCCTAGTTGGGGGGGTGAAGATCCTTTTTGATCCTCT	RV for construction of *in vitro* target DNA with mismatch at PAM distal position 19-20
	BG10587	CATGAGAGGCGCAAAAAGGATCTTCACCCCCCCAACTAGG	FW for construction of *in vitro* target DNA with mismatch at PAM distal positions 17-20
	BG10588	GATCCCTAGTTGGGGGGGTGAAGATCCTTTTTGCGCCTCT	RV for construction of *in vitro* target DNA with mismatch at PAM distal position 17-20
	BG10589	CATGAGAGGCGACAAAAGGATCTTCACCCCCCCAACTAGG	FW for construction of *in vitro* target DNA with mismatch at PAM distal positions 15-20

	Oligo	Sequence	Description
***In vitro* ThermoCas9 Mismatch assay**	BG10590	GATCCCTAGTTGGGGGGGTGAAGATCCTTTTGTCGCCTCT	RV for construction of *in vitro* target DNA with mismatch at PAM distal position 15-20
	BG10591	CATGAGAGGCGACCCAAGGATCTTCACCCCCCCAACTAGG	FW for construction of *in vitro* target DNA with mismatch at PAM distal positions 13-20
	BG10592	GATCCCTAGTTGGGGGGGTGAAGATCCTTGGGTCGCCTCT	RV for construction of *in vitro* target DNA with mismatch at PAM distal position 13-20
	BG10593	CATGAGAGGCGACCCCCGGATCTTCACCCCCCCAACTAGG	FW for construction of *in vitro* target DNA with mismatch at PAM distal positions 11-20
	BG10594	GATCCCTAGTTGGGGGGGTGAAGATCCGGGGGTCGCCTCT	RV for construction of *in vitro* target DNA with mismatch at PAM distal position 11-20
	BG10595	CATGAGAGGCGACCCCCTTATCTTCACCCCCCCAACTAGG	FW for construction of *in vitro* target DNA with mismatch at PAM distal position 9-20
	BG10596	GATCCCTAGTTGGGGGGGTGAAGATAAGGGGGTCGCCTCT	RV for construction of *in vitro* target DNA with mismatch at PAM distal position 9-20
	BG10597	CATGAGAGGCGACCCCCTTCGAGTCACCCCCCCAACTAGG	FW for construction of *in vitro* target DNA with mismatch at PAM distal position 5-20
	BG10598	GATCCCTAGTTGGGGGGGTGACTCGAAGGGGGTCGCCTCT	RV for construction of *in vitro* target DNA with mismatch at PAM distal position 5-20
	BG10599	CATGAGAGGCGACCCCCTTCGAGGACACCCCCCAACTAGG	FW for construction of *in vitro* target DNA with mismatch at PAM distal position 0-20
	BG10600	GATCCCTAGTTGGGGGGTGTCCTCGAAGGGGGTCGCCTCT	FW for construction of *in vitro* target DNA with mismatch at PAM distal position 0-20

Restriction sites are shown in italics. The PAMs are colored red. Spacer regions are shown in bold. Nucleotides in lowercase letters correspond to primer overhangs for HiFi DNA Assembly. LIC: Ligase Independent cloning; FW: Forward primer; RV: Reverse primer.

Supplementary Table 3 | Plasmids used in this study, related to Figures 1 to 4.

Plasmid	Description	Restriction sites used	Primers	Source
pNW33n	*E. coli-Bacillus* shuttle vector, cloning vector, Cam^R	-	-	BGSC
pUC57_T7sgRNAfull	pUC57 vector containing DNA encoding the sgRNA under the control of T7 promoter; serves as a template for *in vitro* transcription of full length Repeat/Antirepeat sgRNAs			Baseclear
pMA2_T7sgRNAtruncated R/AR	Vector containing DNA encoding the truncated Repeat/Antirepeat part of the sgRNA under the control of T7 promoter; serves as a template for *in vitro* transcription of truncated Repeat/Antirepeat sgRNAs	-	-	Gen9
pRARE	T7 RNA polymerase based expression vector, Kan^R	-	-	EMD Millipore
pML-1B	*E. coli* Rosetta™ (DE3) plasmid, encodes rare tRNAs, Cam^R	-	-	Macrolab, Addgene
pEMG	*P. putida* suicide vector, used as template for replicon and Kan^R		See table S1	1
pSW_I-SceI	*P. putida* vector containing *I-SceI*, used as template for *xylS* and P_{Pm}		See table S1	1
pWUR_Cas9sp1_hr	pNW33n with spCas9-module containing spacer targeting the *pyrF* gene. This plasmid was used as a template for constructing the ThermoCas9 based constructs	-	-	2
pThermo_Cas9	*thermocas9* with N-term. His-tag and TEV cleavage site in pML-1B. Expression vector for ThermoCas9	SspI and Ligase Independent Cloning	BG7886 and BG7887	This study
pThermo_dCas9	cas9*dthermo*cas9 with N-term. His-tag and TEV cleavage site in pML-1B. Expression vector for catalytically inactive (dead) dThermoCas9	SspI and Ligase Independent Cloning	BG7886 and BG7888	This study

Plasmid	Description	Restriction sites used	Primers	Source
pNW-PAM7nt	Target sequence in pNW33n vector containing a 7-nt degenerate PAM for *in vitro* PAM determination assay	BamHI and BspHI	See table S1	This study
pNW63-pNW78	Target sequence in pNW33n vector containing distinct nucleotides at the 6th and 7th positions of the PAM (CCCCCNNA)	BamHI and BspHI	See table S1	This study
pThermoCas9_ctrl	pNW33n with ThermoCas9-module[1] containing a non-targeting spacer. Used as a negative control	-	See table S1	This study
pThermoCas9_bsΔpyrF1	pNW33n with ThermoCas9-module[1] containing spacer 1 targeting the *pyrF* gene and the fused us+ds *pyrF*-flanks	-	See table S1	This study
pThermoCas9_bsΔpyrF2	pNW33n with ThermoCas9-module[1] containing spacer 2 targeting the *pyrF* gene and the fused us+ds *pyrF*-flanks	-	See table S1	This study
pThermoCas9i_ctrl	pNW33n with Thermo-dCas9-module[2] containing a non-targeting spacer. Used as a wild-type control	-	See table S1	This study
pThermoCas9i_ldhL	pNW33n with Thermo-dCas9-module[2] containing spacer 2 targeting the *ldhL* gene	-	See table S1	This study
pThermoCas9_ppΔpyrF	pEMG with ThermoCas9-module[3] for *Pseudomonas putida* containing a spacer targeting the a spacer targeting the *pyrF* gene and the fused us+ds *pyrF*-flanks	-	See table S1	This study

[1] The ThermoCas9 module contains *thermocas9* under the native P_{xylL} promoter followed by the sgRNA under the *B. coagulans* P_{pta} promoter (Figure 4).

[2] Like the ThermoCas9 module, but with the *thermo-dCas9* instead of *thermocas9* (Figure 4).

[3] The ThermoCas9 module for *Pseudomonas putida* contains *thermocas9* under the transcriptional control of the inducible Pm-XylS system followed by the sgRNA under the constitutive P3 promoter.

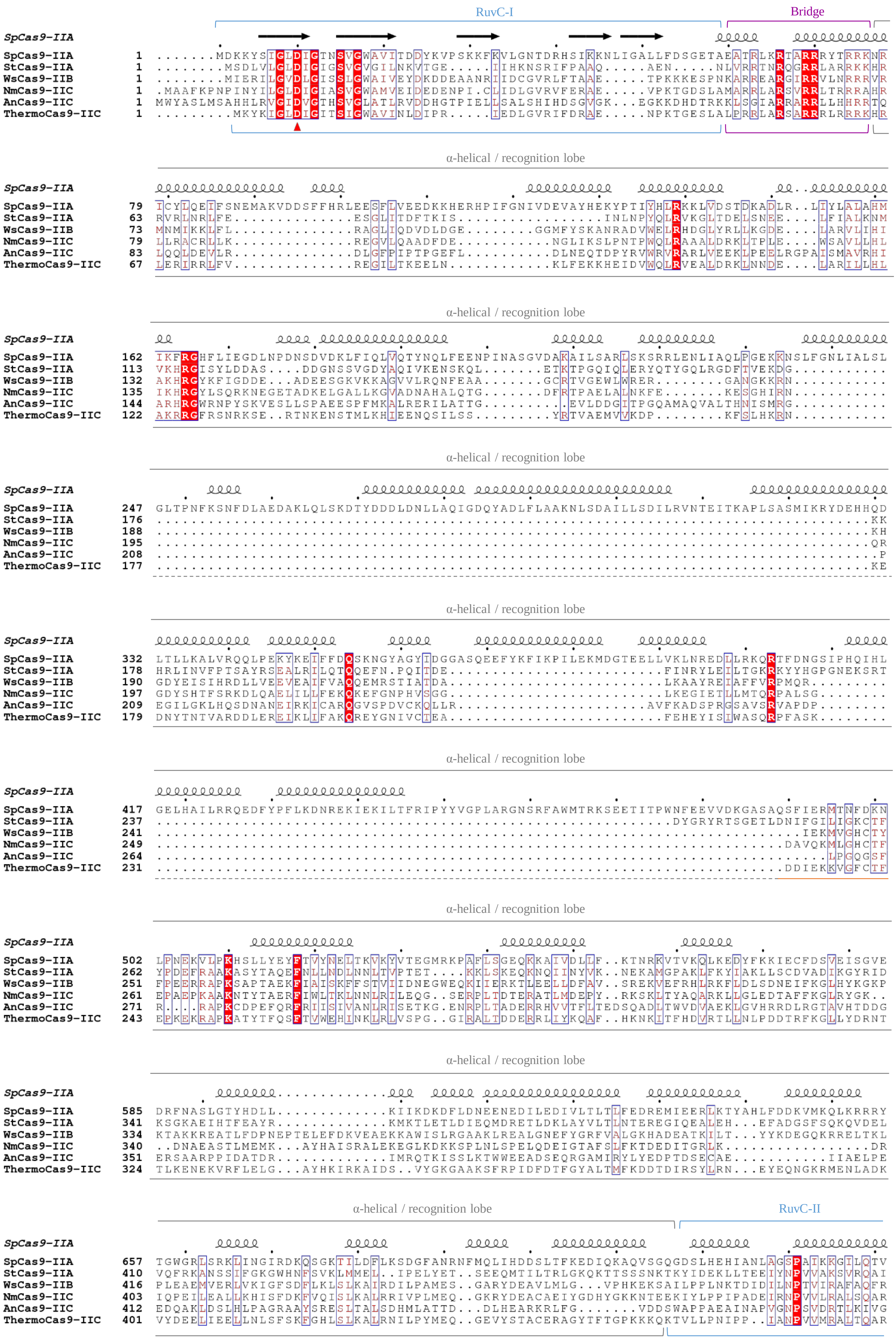
RuvC-I
Bridge
α-helical / recognition lobe
RuvC-II
SpCas9-IIA
StCas9-IIA
WsCas9-IIB
NmCas9-IIC
AnCas9-IIC
ThermoCas9-IIC

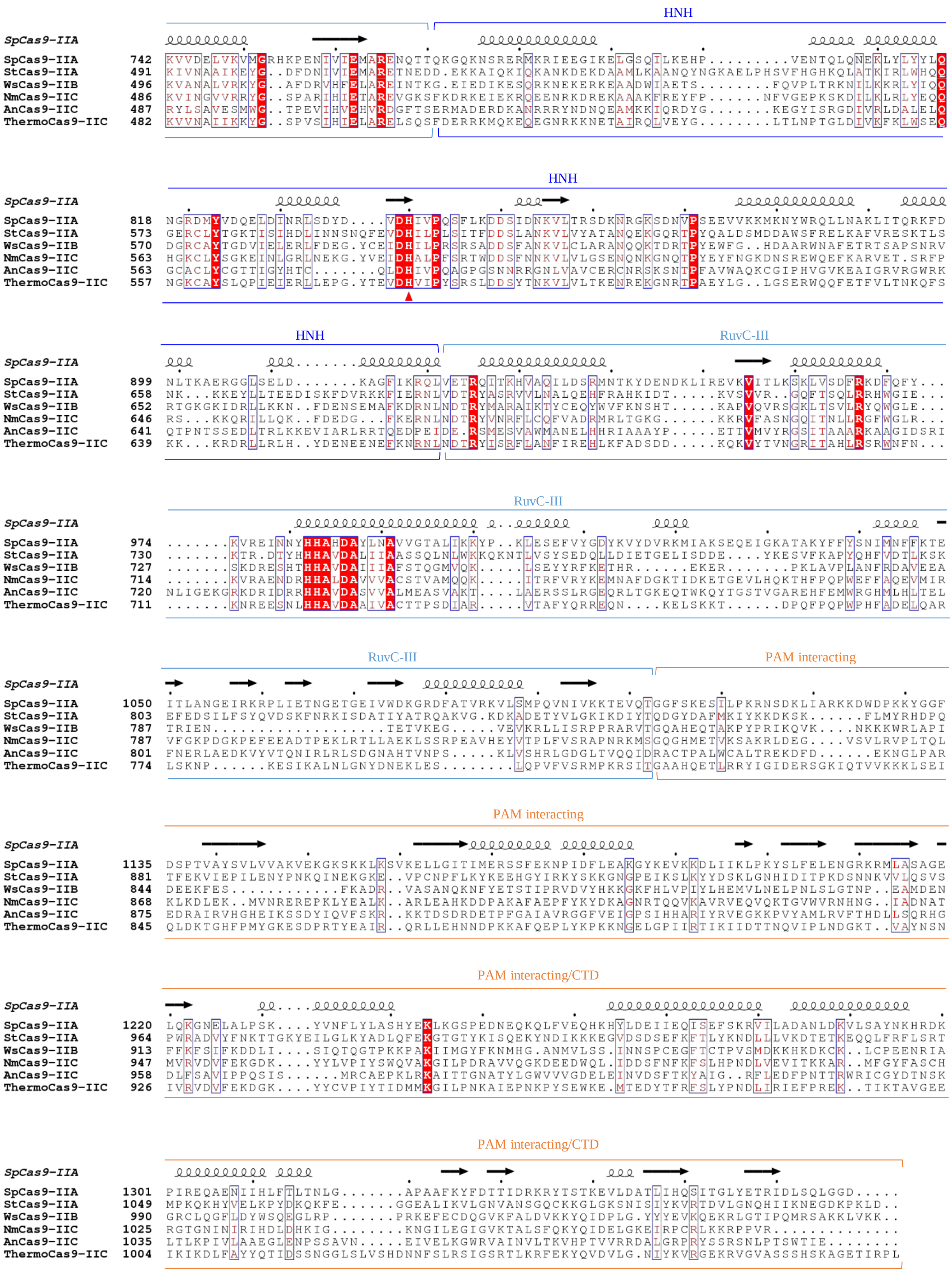

Supplementary Fig. 1 | Multiple sequence alignment of Type II-A, B and C Cas9 orthologues.
Cas9 protein sequences of *Streptococcus pyogenes* (Sp), *Streptococcus thermophilus* (St), *Wolinella succinogenes* (Ws), *Neisseria meningitides* (Nm), *Actinomyces naeslundii* (An), and *Geobacillus thermodenitrificans* (Thermo) were aligned using ClustalW[3] in MEGA7[4] with default settings; ESPript[5] was used to generate the visualization. Strictly conserved residues are shown in white text on red background; similar residues are shown in red text on white background. Red pyramids indicate the two conserved nuclease domains in all sequences. Horizontal blacks arrow and curls indicate β-strands and α-helices, respectively, in the SpCas9 secondary structure (protein database nr 4CMP[6]). Structural domains are indicated for SpCas9 and ThermoCas9 using the same colour scheme as in Figure 1A.

A.

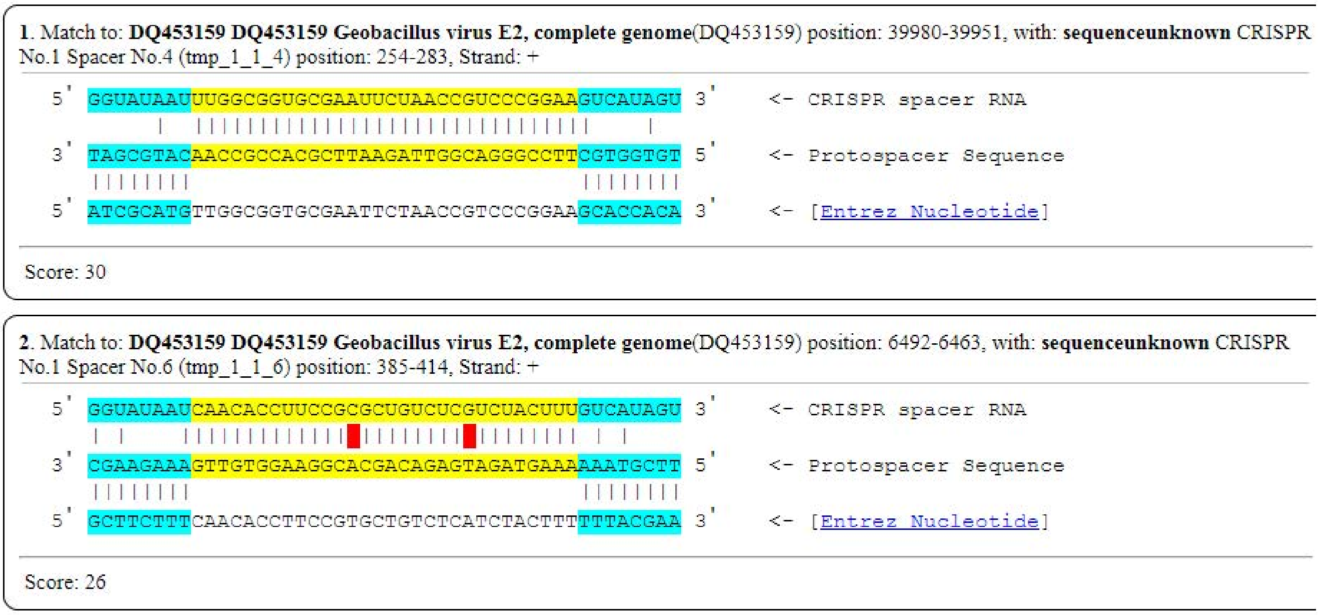

B.

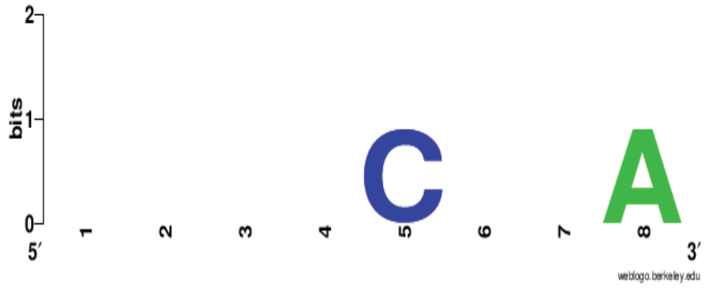

Supplementary Fig. 2 | *In silico* PAM determination results.
(A) The two hits obtained with phage genomes using CRISPRtarget[7].
(B) Sequence logo of the consensus 7-nt long PAM of ThermoCas9, obtained by in silico PAM analysis. Letter height at each position is measured by information content[8].

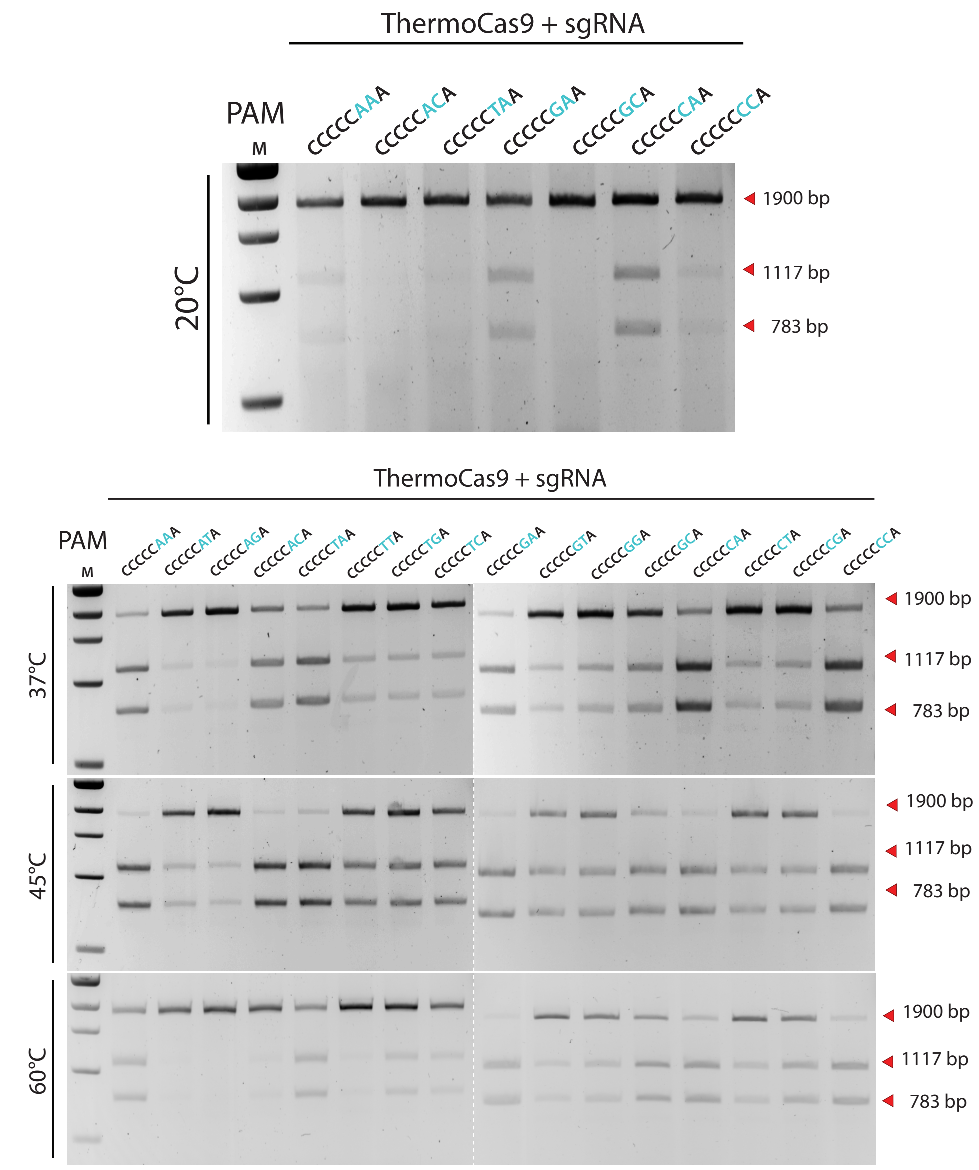

Supplementary Fig. 3 | ThermoCas9 PAM discovery.
In vitro cleavage assays for DNA targets with different PAMs at 20˚C, 37˚C, 45˚C and 60˚C. Seven (20˚C) or sixteen (37˚C, 45˚C, 60˚C) linearized plasmid targets, each containing a distinct 5'-CCCCCNNA-3' PAM, were incubated with ThermoCas9 and sgRNA, then analysed by agarose gel electrophoresis.

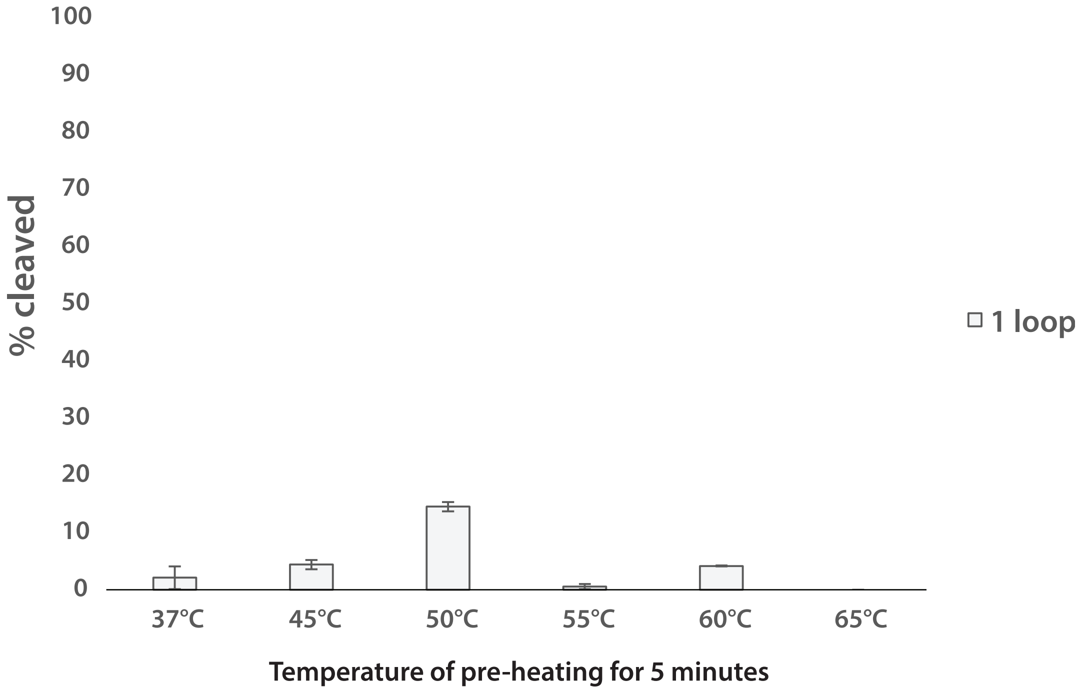

Supplementary Fig. 4 | Activity of ThermoCas9 at a wide temperature range using sgRNA containing one stem-loop.
The importance of the predicted three stem loops of the tracrRNA scaffold was tested by transcribing truncated variations of the sgRNA and evaluating their ability to guide ThermoCas9 to cleave target DNA at various temperatures. Shown above is the effect of one stem-loop on the activity of ThermoCas9 at various temperatures. Average values from at least two biological replicates are shown, with error bars representing S.D.

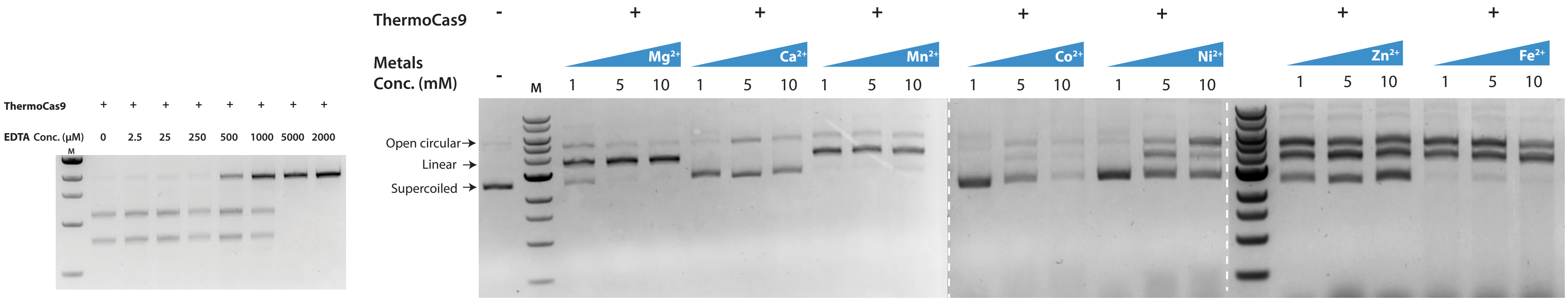

B.

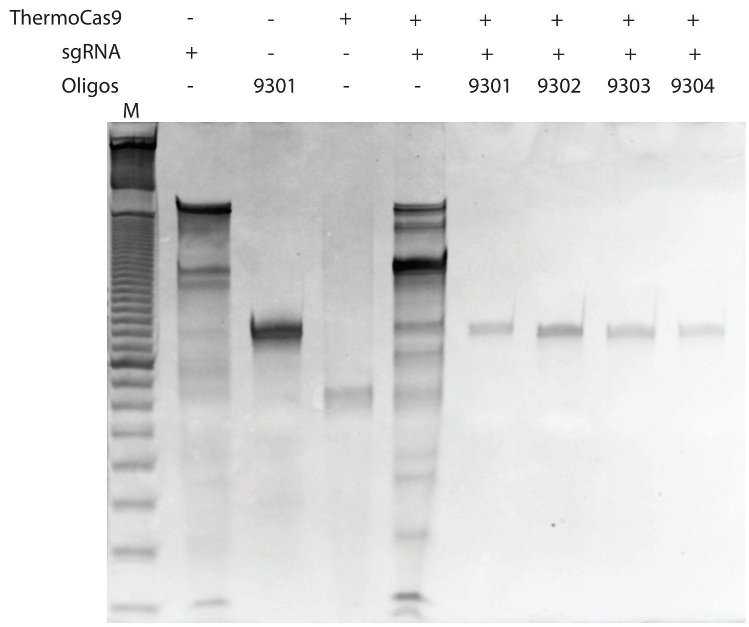

Supplementary Fig. 5 | ThermoCas9 mediates dsDNA targeting using divalent cations as catalysts and does not cleave ssDNA.
(A) *In vitro* DNA cleavage by ThermoCas9 with EDTA and various metal ions. M = 1 kb DNA ladder.
(B) Activity of ThermoCas9 on ssDNA substrates. M= 10 bp DNA ladder.

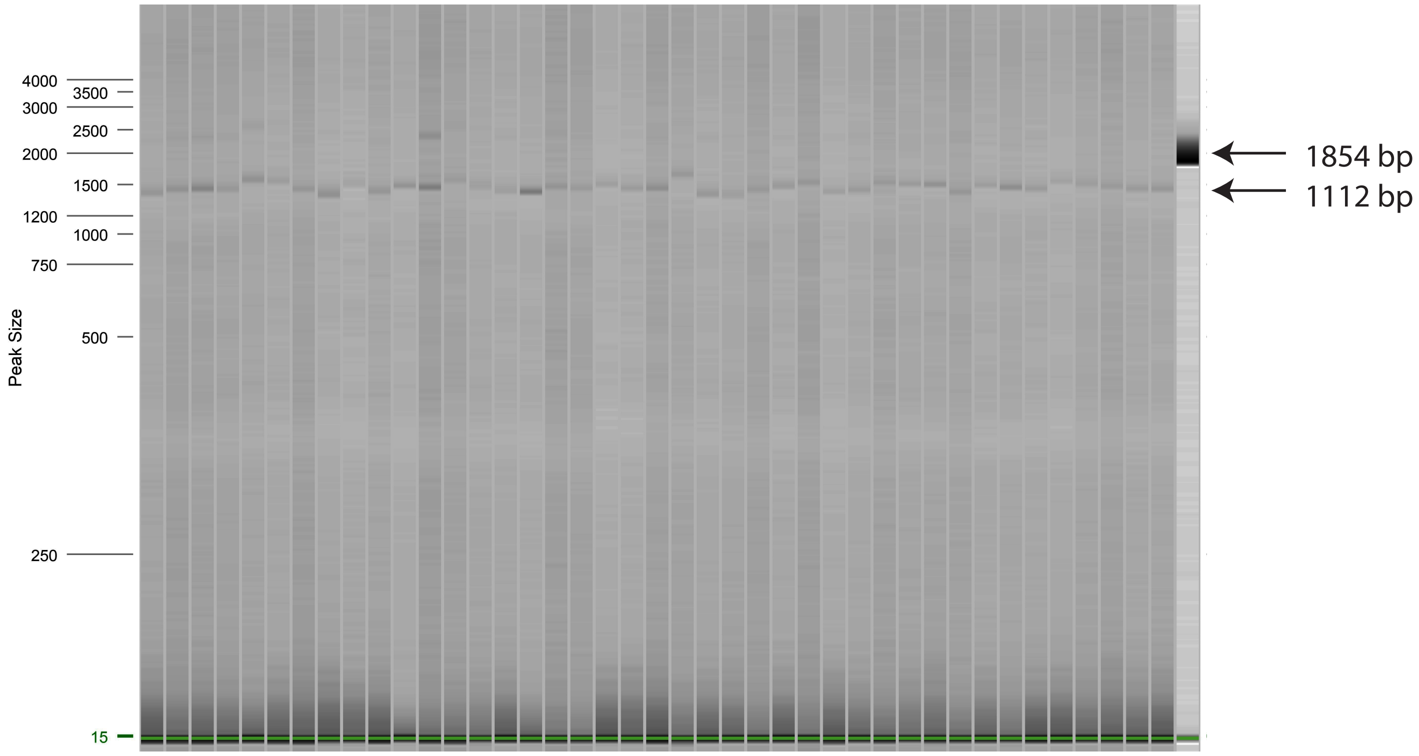

Supplementary Fig. 6 | Colony PCR of *P. putida* ThermoCas9-based *pyrF* deletion.
Capillary gel electrophoresis showing the resulting products from genome-specific PCR on the obtained colonies from the ThermoCas9-based *pyrF* deletion process from the genome of *Pseudomonas putida.* The 1854 bp band and the 1112 bp band corresponds to the *pyrF* and Δ*pyrF* genotype, respectively.

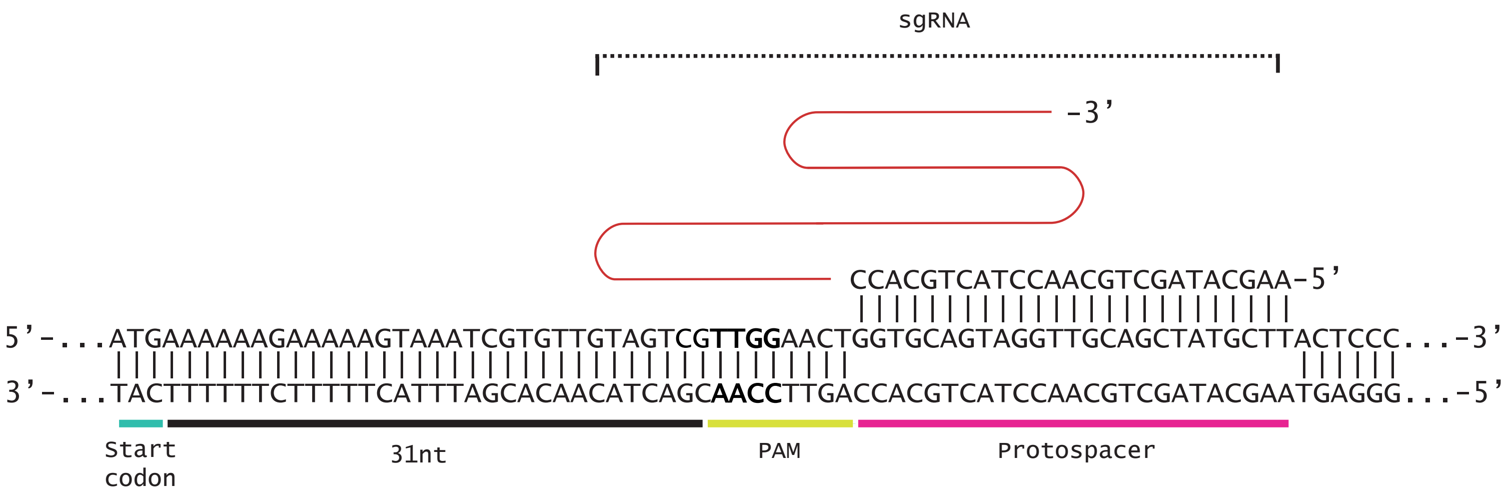

Supplementary Fig. 7 | Spacer selection for the *ldhL* silencing experiment.
Schematic representation of the spacer (sgRNA)-protospacer annealing during the *ldhL* silencing process; the selected protospacer resides on the non-template strand and 39nt downstream the start codon of the *ldhL* gene. The PAM sequence is shown in bold.

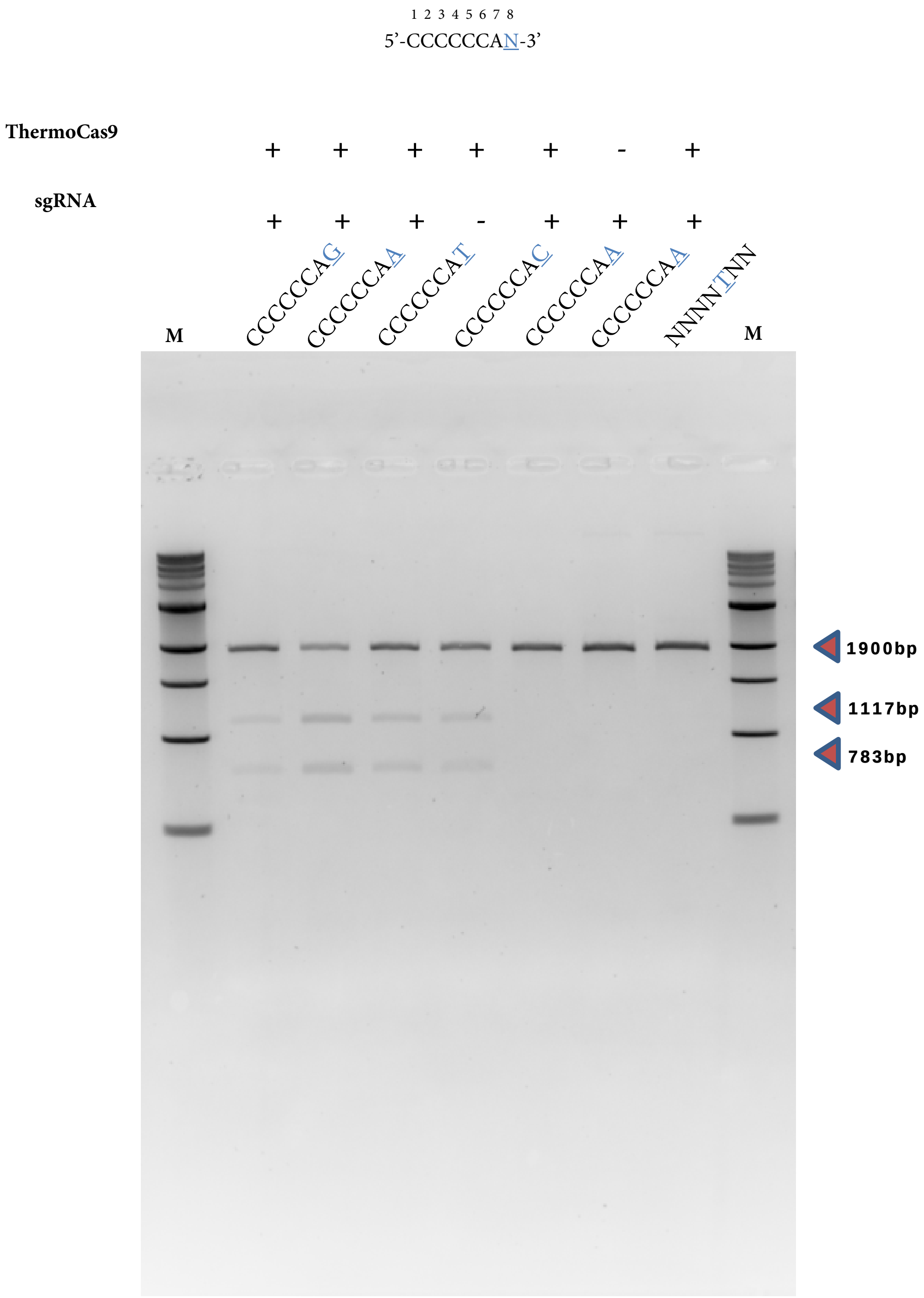

Supplementary Fig. 8 | Uncropped blots of figure 2c.

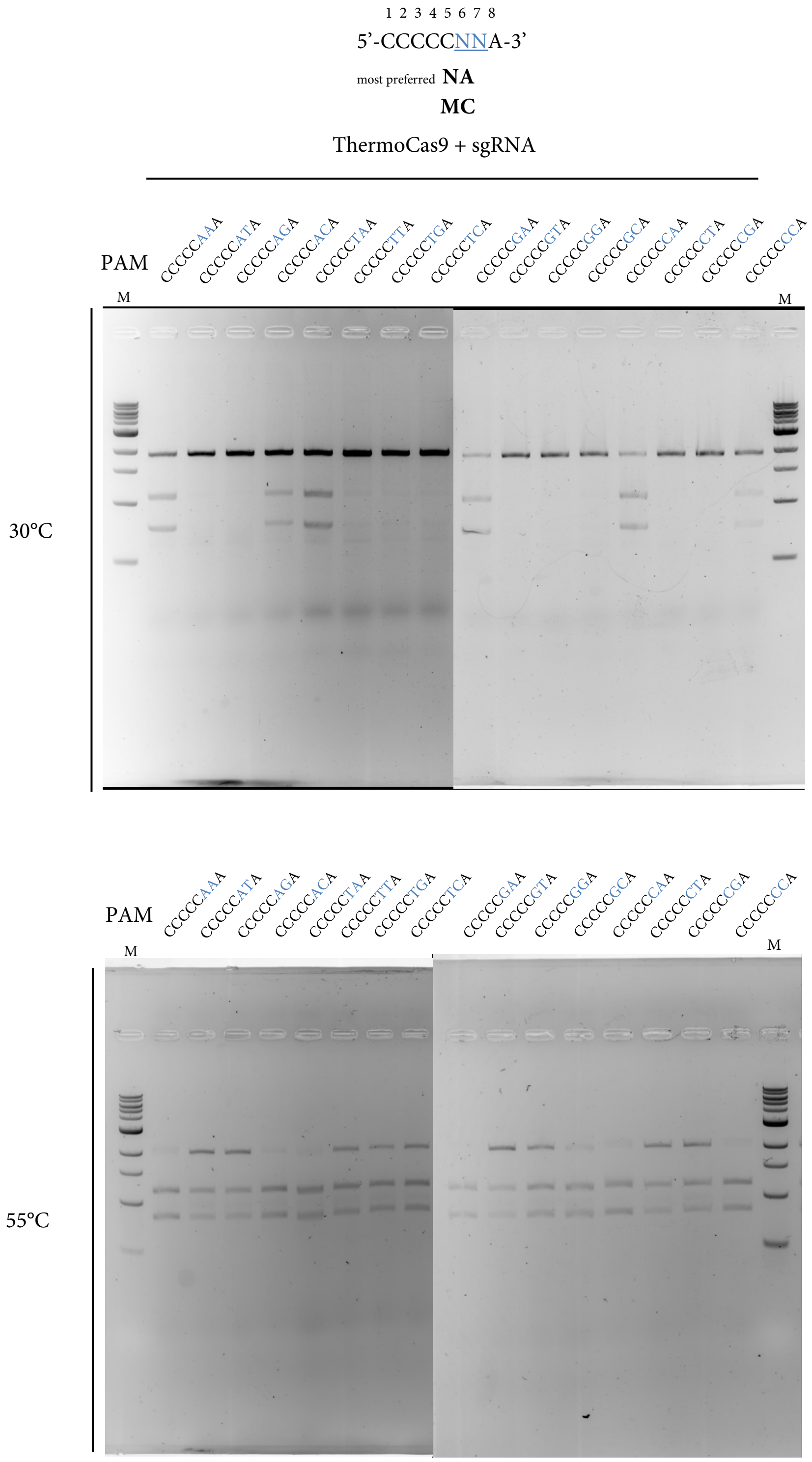

Supplementary Fig. 9 | Uncropped blots of figure 2d.

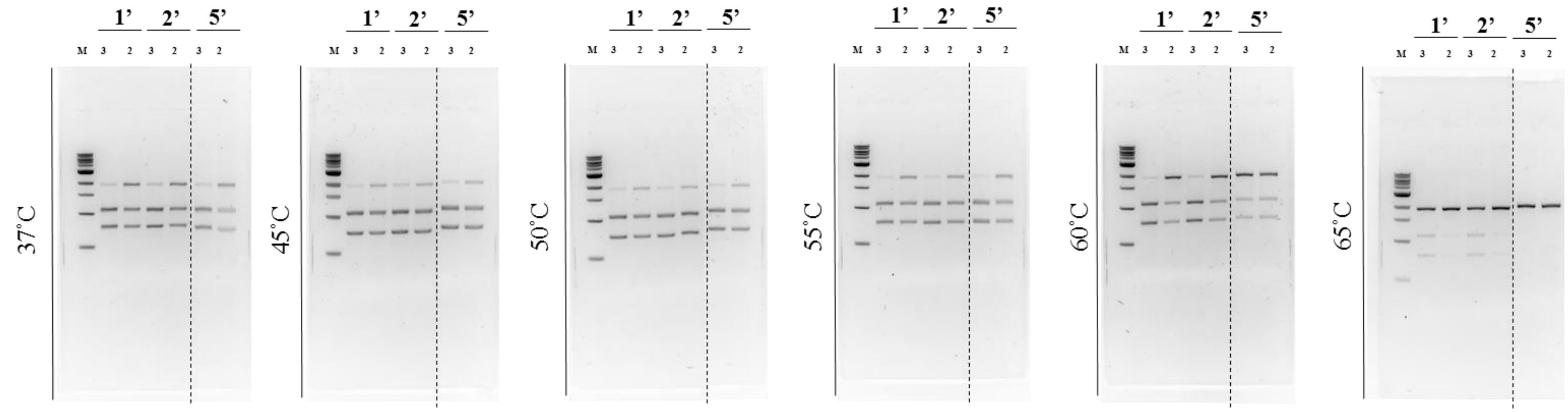

Supplementary Fig. 10 | Agarose gels used for quantification of cleavage using 3- and 2- hairpins in the sgRNA as shown in figure 3b.

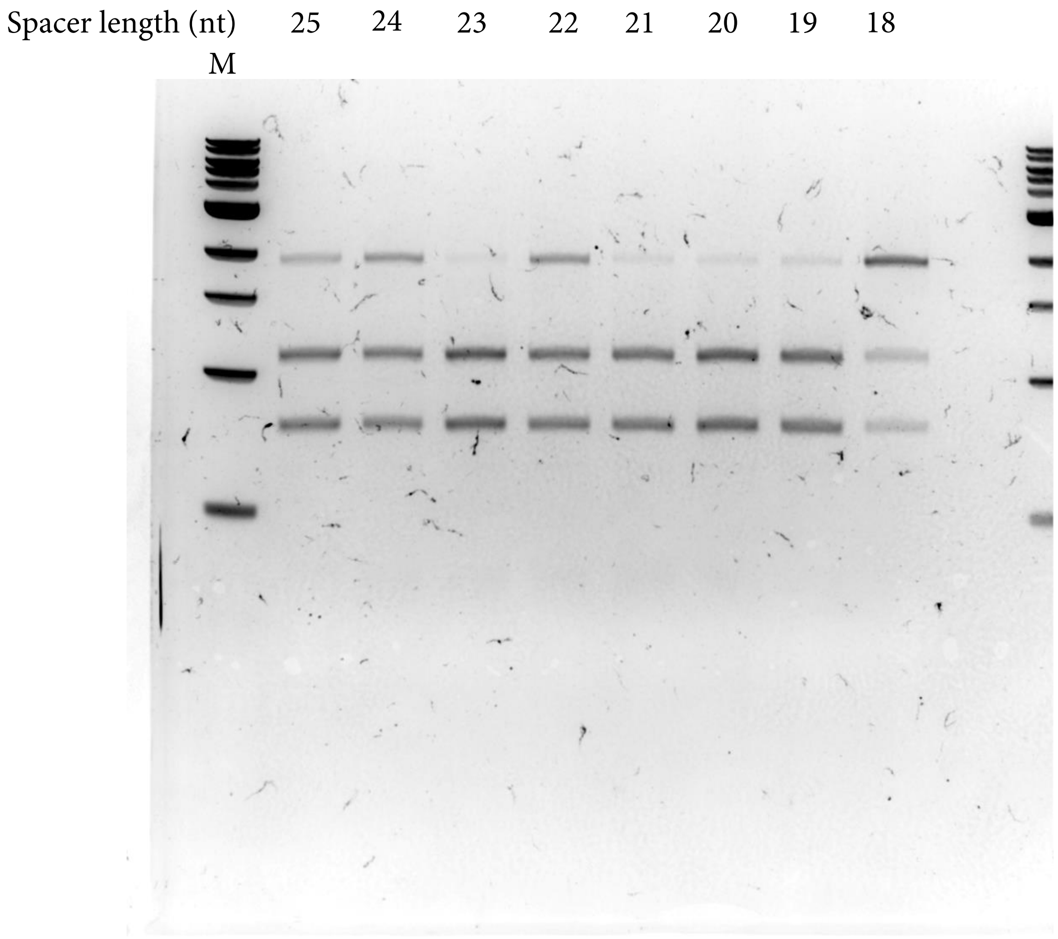

Supplementary Fig. 11 | Uncropped blots used for the quantification of spacer length truncations assays in figure 3c.

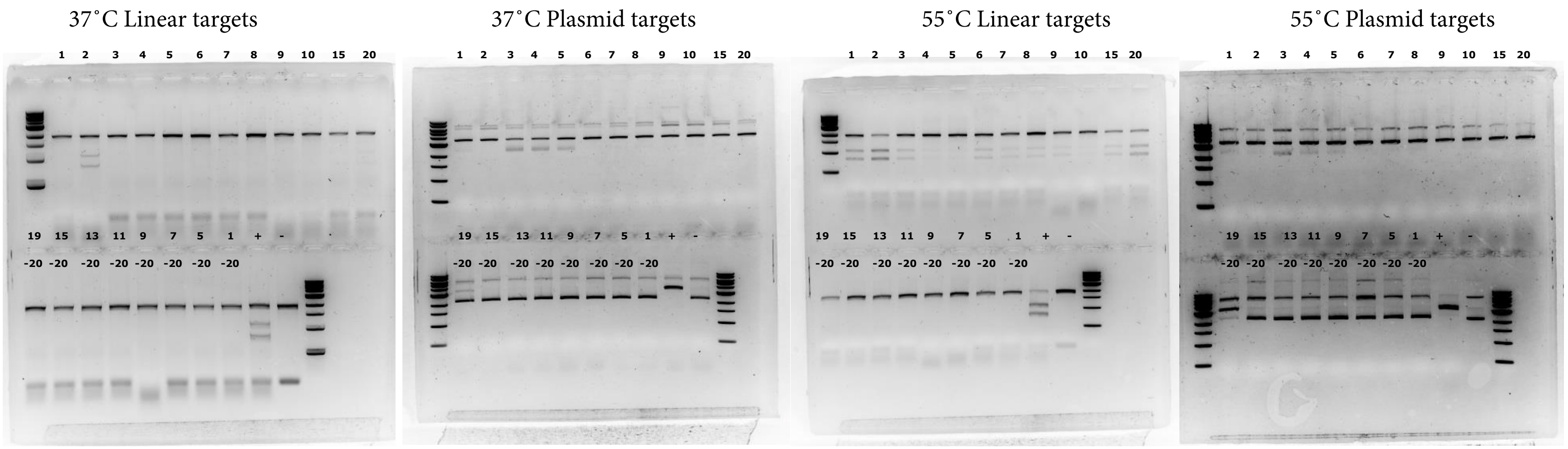

Supplementary Fig. 12 | Agarose gels used for the quantification of mismatch tolerance assay in figure 4b, c.

Colony PCR: *Bacillus smithii* Δ*pyrF*

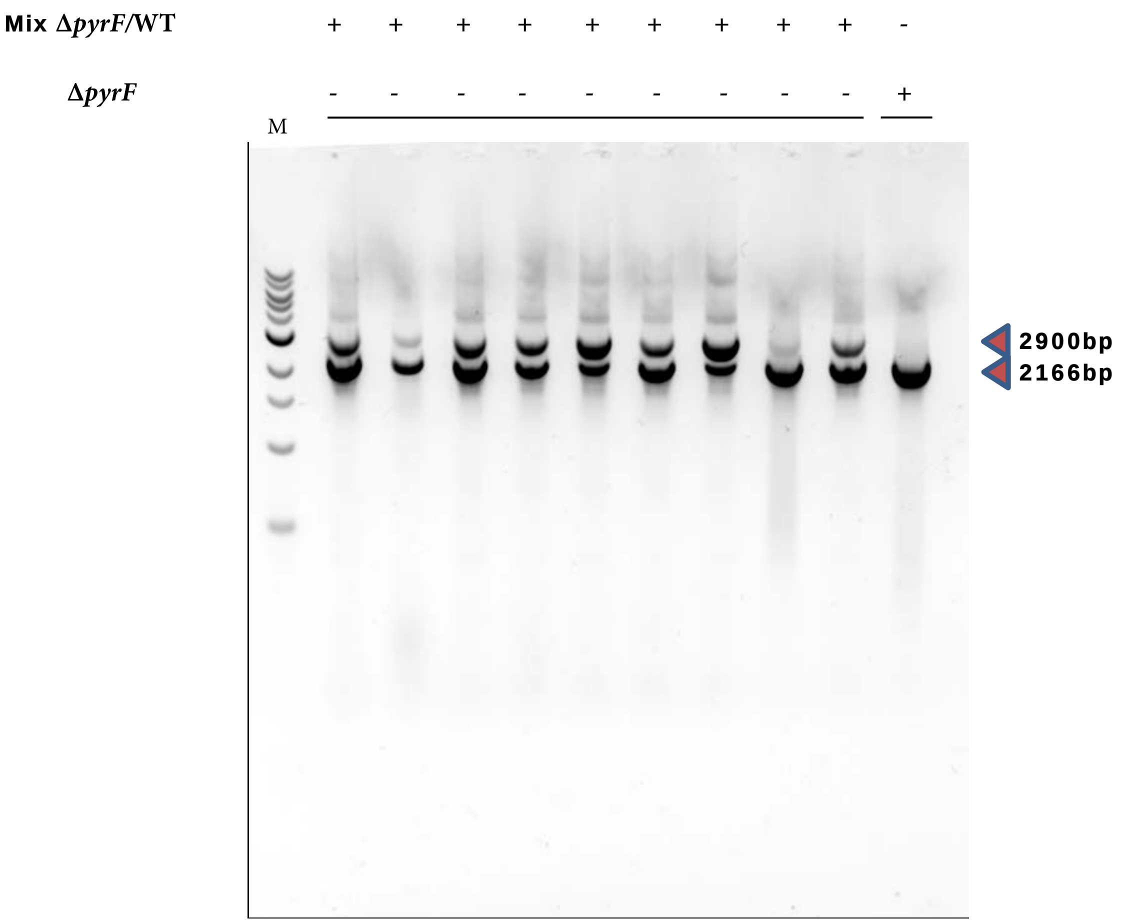

Supplementary Fig. 13 | Uncropped blot of figure 5b.

Supplementary references

1. Martinez-Garcia, E. & de Lorenzo, V. Engineering multiple genomic deletions in Gram-negative bacteria: analysis of the multi-resistant antibiotic profile of Pseudomonas putida KT2440. *Environ. Microbiol.* **13,** 2702–2716 (2011).

2. Mougiakos, I. *et al.* Efficient genome editing of a facultative thermophile using mesophilic spCas9. *ACS Synth. Biol.* **6,** 849–861 (2017).

3. Larkin, M. A. *et al.* Clustal W and Clustal X version 2.0. *Bioinformatics* **23,** 2947–2948 (2007).

4. Kumar, S., Stecher, G. & Tamura, K. MEGA7: Molecular evolutionary genetics analysis version 7.0 for bigger datasets. *Mol. Biol. Evol.* **33,** 1870–1874 (2016).

5. Gouet, P., Courcelle, E., Stuart, D. I. & Métoz, F. ESPript: analysis of multiple sequence alignments in PostScript. *Bioinformatics* **15,** 305–8 (1999).

6. Jinek, M. *et al.* Structures of Cas9 endonucleases reveal RNA-mediated conformational activation. *Science (80-.).* **343,** 1247997–1247997 (2014).

7. Biswas, A., Gagnon, J. N., Brouns, S. J. J., Fineran, P. C. & Brown, C. M. CRISPRTarget: bioinformatic prediction and analysis of crRNA targets. *RNA Biol.* **10,** 817–827 (2013).

8. Crooks, G. E., Hon, G., Chandonia, J.-M. & Brenner, S. E. WebLogo: A sequence logo generator. Genome Res. 14, 1188–1190 (2004).
